# Common Post-fitting Complications in Tooth-supported Fixed-Fixed Design Metal-Ceramic Fixed Dental Prostheses

**DOI:** 10.12669/pjms.303.5599

**Published:** 2014

**Authors:** Nadia Zafar, Fazal Ghani

**Affiliations:** 1 Nadia Zafar, BDS, Department of Prosthodontics, Khyber College of Dentistry, University Campus, Peshawar, Pakistan.; 2 Fazal Ghani, BSc, BDS, MSc, CMP, PhD, FDSRCPSGlasg, Department of Prosthodontics, Khyber College of Dentistry, University Campus, Peshawar, Pakistan.

**Keywords:** Metal-ceramic fixed dental prostheses, Complications, Fixed dental prostheses survival

## Abstract

**Objectives: **To report the frequency of common complications and their levels in metal-ceramic fixed dental prostheses (MC-FDPs).

**Methods: **A Descriptive Cross-sectional study was conducted at the Prosthodontics Department, Khyber College of Dentistry Peshawar from January 2011 to October 2012. Using a structured proforma, data from 139 subjects fulfilling the inclusion and exclusion criteria for the study and reporting complications in their MC- FDPs were collected using the method of interview, clinical & radiographic examination.

**Results:** Of 139 subjects (Mean age = 34+ 6.4 Years), 81 (58.3%) were males and 58 (41.7%) were females with a male to female ratio of 1.4:1. De-cementation was the most common complication (41.7%). Least common complication was secondary caries (6.5%). Level-1 complications were more prevalent (77.7%) than level-2 complications (22.3%). In 91.4% cases, complications occurred before the FDP completed their fifth-years’ service life with 25.2% of these occurring within the first years’ service life.

**Conclusion:** Irrespective of the type of complications, level-1 complications were more common with de-cementation being the most common complication. One-quarter of all the complications occurred within the first-year service life of the FDPs highlighting concern over the quality of the provided MC-FDPs. .

## Introduction

Tooth-supported fixed dental prostheses (FDP) will remain the first choice treatment for restoring patients with partial edentulism.^1^ Among the different types of FDPs available, those using the metal-ceramic technology have been the standard for replacement of missing teeth for more than 60 years.^2^ This is attributed to the natural looking overlying ceramic supported by high strength metal alloy core underneath. Added to this property is its ease of fabrication and affordable cost.^3^These prostheses have been recognized as an essential treatment entity of prosthodontics practices globally. Burke et al ^4^ reported that in a period of 10 years of the total of 47474 crowns placed by the NHS general dental practitioners in England and Wales, 80% utilized the metal-ceramic (MC) technology. 

To know about the functional performance of metal-ceramic FDPs, extensive research work has been directed to evaluate their success and survival rates. Studies of the kind on longevity of FDPs have shown maximum survival rates of 98% after 5 years, 97% after 10 years and 85% after 15 years respectively.^5^ A retrospective study conducted in Singapore illustrated that 58% of FDPs remained successful and required no intervention over a post-fitting functional period of 5 years.^6^ Another study carried out in Japan showed a survival rate of 74% at 15 years.^7^ A study on long term efficacy of FDPs concluded that after 20 years, the survival rate was 66.2%.^8^ All these suggest a decreasing survival rate with an increasing intraoral period of the FDPs. Despite the above reported promising survival data of FDPs, a very high proportion of complications of different categories are frequently encountered.^9^ Eliasson et al ^5^ reported 34% of FDPs with complications. For FDP a mean complication incidence of 27% was calculated while evaluating the prostheses for periods between 5 - 14 years.^10^

Complications in FDPs can be identified as abutment related or biological complications or prostheses related termed as technical complications. Biological complications comprise of secondary caries, pulpal pathology, periodontal disease and abutment fracture. On the other hand, technical complications comprise of loss of retention (de-cementation), ceramic de-bonding, aesthetic problems, occlusal faults and breakage of connectors or prostheses.^11^ A retrospective study of failure and complications in abutments and FDPs in patients showed that some 67.4% abutments suffered from periodontal problems.^7^ Another study reported 61% of FDP complications comprised of secondary caries and loss of retention.^8^ Oginni^12^ found poor aesthetics in 40.5% as a common complication. In a study by Napankangas et al^13^ceramic de-bonding (17.6%) was a major complication. A systematic review estimated risk of FDP complication because of loss of pulp vitality as 10% over a 10 years period.^14^ It is evident that the occurrence of each of the complications is very much varied indicating concern and putting to questioning the performance and abilities of all including clinicians, technicians and patients. 

The so reported frequent occurrence of FDP complications and the very scarce local research work suggests continuation of work in this area. This would help in our understanding of the level of severity and causes of complications that may range from those that are easily correctable without FDP replacement to those requiring their redesigning and replacement.^15^

## Methods

A descriptive, cross-sectional study of 22-months duration from January 2011 to October 2012 at the Prosthodontics Department of Khyber College of Dentistry, Peshawar was conducted. A total of 139 subjects were included in the study fulfilling the inclusion and exclusion criteria. The subjects were those having complications in their MC-FDPs and their supporting teeth (abutments). To control confounders and avoid introducing bias in the results, a consecutive (non-probability) sampling technique the following criteria for the including study participants were considered; adults from both genders with age range 20-40 years and wearing MC-FDP of fixed-fixed design having full coverage retainers and supported by natural teeth as abutments and having problems related to their MC-FDPs or abutments. Patients having complications in FDPs other than MCFDPs and those because of blow or trauma to the mouth and FDP were not considered.

Data for only common complications was collected. For the purpose of this study, a complication referred to a condition considered unwanted or unacceptable to both the patient and the clinician and that it has occurred after the provision of FDP. The following two main types of complications were considered:

- Abutment related biological complications: These included secondary carries and / or pulp pathology and periodontal disease.
- FDP related technical complications: These included FDP de-cementation and unacceptable aesthetics with the FDP.

The level of each complication was also documented to indicate either the extent of progression of an individual complication in case of abutment related biological or the need for the easiness / difficulty in repair or the need for replacement work in case of a technical complication. The service life rendered by the MC-FDP was categorized as; less than 1-year, from 1-5 years and from 6-10 years. Each of the complications was categorized into two levels. 

**Secondary Caries and /or Pulp Pathology:** On examination of the abutment retaining and supporting the FDP, with the FDP in situ or removed it exhibits secondary caries and /or pulp pathology of;

***Level-1:*** Presence of white / black spots to cavity (detected by clinical examination), feeling of pain / sensitivity, abutment tender on percussion and / or radiographic evidence for presence of pathology around the root apex of the tooth. With the provision of necessary treatment the abutment can be saved and used.

***Level- 2:*** On examination, abutment exhibits grossly carious lesion deep under the gingiva and / or presence of swelling or sinus / fistula formation with advanced peri-apical tissue damage (detected by clinical examination and radiograph) requiring the extraction of abutment.

**Periodontal Disease:**

***Level-1:*** Gingival inflammation (detected clinically), loss of alveolar bone (detected by radiograph) and formation of pockets (detected by probing). It is possible to restore the periodontal health of the abutment.

***Level-2:*** Excessive mobility of abutment (detected clinically). The abutment is untreatable and unable to provide support to the same FDP. 

**De-cementation:**

***Level-1:*** When it occurred either due to excessive dislodgement forces because of correctable occlusal errors and/or during normal chewing.

***Level-2:*** If in addition to de-cementation, the dislodged FDP has un-correctable occlusal errors, abutment has sustained and / or fracture that cannot be corrected for reuse of FDP support.

**Unacceptable aesthetics:**

***Level-1:*** Patient feels shade mismatch however the clinician considers it of minor problem without the need for replacing the FDP.

***Level-2:*** Bulky restoration, inadequate anatomical form / shape, metal core visible (inadequate porcelain thickness) and loss / chipping of ceramic facing (detected by clinical examination). Either redesigning / remaking of FDP are required or the use of a conventional FDP is excluded.

The institutional evaluation unit (REU) approved the study. Subjects reporting with FDP complications and who fulfilled the inclusion criteria were invited to participate. Data were collected using structured proforma. Relevant history was recorded and considering the subjects reason for consultation, the nature of complication and its level was determined. Detailed intra-oral examination was also carried out using standard techniques of inspection, palpation, percussion and probing along with radiographic examination. Prostheses were evaluated for number of units, retainers, pontics along with associated complications including de-cementation and unacceptable aesthetics. Complications associated with abutment such as secondary caries and / or pulp pathology and periodontal disease were also noted. 

The collected data were computed, tabulated and analysed using SPSS version 16. Descriptive statistics like mean ± standard deviation (SD) for the age and service life of the FDP and frequencies for number of units, number of pontics in the FDP at the time of presented complication(s) were done. Similarly the proportions were calculated for the categorical variables like FDPs location in the arches, status of abutment teeth, and the type of complication (abutment or FDP related). FDP complications and their levels were stratified among the age, sex, location of FDP, to see the effect modifiers. Male to female ratio was calculated for gender distribution. 

## Results

Subjects were distributed as males (81, 58.3%) and females (58, 41.7%) with male to female ratio of 1.4:1. The mean age in years of the subjects was 34±6.4 (S. Dev). Majority (74.8%) of the subjects with complications in their MC-FDPs were above the age of 30 yrs. Complications were predominant (74.8%) in the FDPs of the subjects belonging to the age group of 31-40 years as compared to 25.2% FDP complications in those belonging to the age group of 20-30 years. 

Among the complications seen in the FDPs, 53 (39%) were abutment related as to 86 (62%) related to technical complications within the FDPs themselves. The data for the numbers of units and pontics in the FDPs with complications are given in Table 1 & 2 respectively. A large proportion of the abutments (n= 286, 85.5%) used for support of FDPs were structurally intact as compared to 14 ((4.3%) having intra-coronal restorations with another 33 (9.9%) having received prior endodontic therapy. 

Data for the service life rendered at the time of occurrence of complications in the FDPs is given in Table 3. Similarly**, t**he arch-wise distribution of the FDPs having complications and of the distribution of various types of complication and their levels are detailed Table- 4. 

**Table 1:** Distribution of No. of units in FDPs

*Units in FDPs*	*Maxilla No. ( %)*	*Mandible No. ( %)*	*Total* *No. (%)*
3	36 (50.7)	45 (66.2)	81(58.3)
4	10 (14.1)	12 (17.6)	22 (15.6)
5	11 (15.5)	05 (07.4)	16 (11.5)
6	05 (07.4)	05 (07.4)	10 (07.2)
7	02 (02.8)	00 (00.0)	02 (01.4)
8	02 (02.8)	00 (00.0)	02 (01.4)
9	02 (02.8)	01 (01.5)	03 (02.2)
10	02 (02.8)	00 (.00)	02 (01.4)
14	01 (01.4)	00 (00.0)	01 (00.7)
Total	71 (51.1)	68 (48.9)	139 (`100)

**Table 2:** Distribution of No. of pontics in FDPs

*No. of pontics*	*Frequency (%)*
1	86 (61.9)
2	35 (25.2)
3	08 (05,8)
4	03 (02.2)
5	06 (04.3)
7	01(00.7)
Total	139 (100)

**Table 3:** Service life rendered by FDPS at time of complication

*Complication*	*FDP No. (%)*			*Total* *No. (%)*
	*< 1-yr*	*1-5 yrs.*	*6-10 yrs.*	
Secondary caries	04(02.9)	05 (03.6)	00 (0.0)	09 (06.5)
Pulpal pathology	3 (02.2)	06 (04.3)	02 (01.4)	11 (07.9)
Periodontal disease	07(05.0)	25 (17.9)	01 (00.7)	33 (23.7)
De-cementation	14(10.1)	38 (27.3)	06 (04.3)	58 (41.7)
Unacceptable esthetics	07(05.0)	18 (12.9)	03 (02.2)	28 (20.2)
Total	35(25.1)	92 (66.1)	12 (08.6	139 (100)

**Table 4:** Arch-wise distribution and location of the FDPS with various complications

Complication	Arch location						Total No. (%)	P value
	Maxilla No. (%)			Mandible No. (%)				
	Anterior	Posterior	Both	Anterior	Posterior	Both		
Secondary caries	0 (00.0)	4 (02.9)	1 (00.7)	0 (00.0)	4 (02.9)	0 (00.0)	9 (06.5)	0.59
Pulp pathology	4 (02.9)	3 (02.2)	1 (00.7)	0 (00.0)	2 (01.4)	1 (00.7)	11(07.9)	0.16
Periodontal disease	5 (03.6)	6 (04.3)	7 (05.0)	0 (00.0)	13 (09.4)	2 (01.4)	33 23.7)	0.63
De-cementation	6 (04.3)	16 (11.5)	8 (05.8)	3 (02.2)	22 (15.8)	3 (02.2)	58 (41.7)	0.30
Unacceptable esthetics	3 (02.2)	4 (02.9)	3 (02.2)	0 (00.0)	18 (12.9)	0 (00.0)	28 (20.2)	0.47
Total	18 (13.0)	33 (23.8)	20(14.4)	3 (02.2)	59 (42.4)	6 (04.3)	139(100)	

Chi square test of proportion (*X*^2 ^=) & Degree of freedom (df): Secondary caries: *X*^2 ^= 3.68, df = 5,

Pulp pathology: *X*^2 ^= 7.89, df = 5, Periodontal disease: *X*^2 ^= 3.46, df = 5, De-cementation: *X*^2 ^= 6.0, df = 5,

Unacceptable esthetics: *X*^2 ^= 9.64,df =10.

## Discussion

Metal-ceramic FDPs used globally have performed satisfactory functions by surviving for a long period (15-20 Years) especially when provided under ideal conditions by dental specialists.^16^ On the other hand the first local studies on FDP complications carried out by Memon and & Ghani ^3^ and Ghani and Memon^11^ in the years 2007 and 2008 revealed that majority of the FDPs had complications within their first 5-years of service life and that the percentage of FDPs free of complications and remaining functional for more than 5 years was as low as 17%. In the present study 91% of the FDPs had complications before completing their 5 years of service life. Thus the situation here locally is not only consistent and not as desired. 

It must be kept in mind that evaluation and comparison of data for studies on FDP longevity and complications is difficult because of several reasons including the use of non-standardized patient population and materials and the patients treated by clinicians with varying skills and experience levels including general dental practitioners, UG and PG dental students, dental specialist other than prosthodontists. The varying levels of skills do affect the outcome. Different workers have used different materials, parameters and criteria for success and failure which makes comparison further difficult. To facilitate valid data for success, it is necessary to conduct a randomized controlled longitudinal study of sufficient duration with well-defined aspects including standardization of tooth preparation parameters, selection of controlled patient population, use of standardized laboratory procedures performed by skilled dental technicians and patient’s education and motivation towards standardized oral hygiene maintenance regime. Despite these reservations and limitation inherent in this study as well as those done in the past, the present study provides data for explaining and comparing in terms of many aspects related to the patients, clinicians and technician’s roles.

To control the confounders, the participants were taken from a specific age group. Thus its findings are to be compared with caution with those of the studies who have not done so. The findings, however, do confirm that FDPs are demanded more by younger subjects. In terms of gender distribution most of the patients who presented complications in their FDPs were males (58.3%). These findings are supported by the study conducted by Ghani and Memon.^11^An international study observed a higher number of females reporting complications in their prostheses as compared to males.^17^ This difference may be due to the fact that females in this part of the world usually are home bound. They do not seek treatment readily for many reasons.^3^ Also females from affluent families do not prefer attendance at the public sector hospitals. This might also have contributed to the lower number of females observed in the present study. Perhaps another likely reason could be the better maintenance of oral hygiene by females wearing FDPs.

More complications (51.1%) were observed in FPDs fitted in the maxillary arch as compared to those in the mandibular arch (48.9%). However, in both arches the incidence of complications was higher in the posterior region of the arches. Majority of the complications were noted in the mandibular posterior region (42.4%) followed by maxillary posterior region (23.7%), maxillary anterior region (12.9%) and mandibular anterior region (2.2%). These findings are in accordance to a previous local study by Ghani and Memon.^3^ On the other hand an international study^18^ reported majority of the FPD complications in the anterior segment of the maxillary arch. This difference of the findings could be explained by the facts that the majority of the FDPs in patients of the present study were fitted in the posterior aspect of the jaws. Furthermore, the nutritional and social habits are varying among patients from different regions. In our region, FDPs are mainly needed by patients for improving the function of mastication. In contrast, in developed countries FDPs are mainly demanded and provided to patients for aesthetic reasons. These explanations were given in a previous local study by Ghani and Memon.^3^ Thus the observation of the finding of more complications in the posterior FDPs in this study as compared to another^18^ is as expected. 

A large number of FDP abutments were structurally intact. It is a common finding that while preparing such abutments, clinicians are over-cautious in order to avoid pulp exposure resulting in inadequate abutment preparation. This may be a contributing factor for de-cementation. Thus the proportion of abutment related complications was much lower than the FDP related complications. Ghani and Memon^11^ pointed out in their study that perhaps less experienced clinicians and most of the quacks or the unqualified practitioners provide FDPs to patients by preferring structurally intact abutments instead of using structurally compromised abutments that could increase the likelihood of FDP failure at an early period of their success life. In the present study, this could have been a possible reason for the lower risk of abutment failure.

In this study most of the complications were those associated with FDPs. These included FDP de-cementation and unacceptable FDP aesthetics. This may suggest problems with the technical and laboratory aspects of fixed prostheses fabrication. Thus the clinicians should involve themselves in the laboratory aspects of FDP manufacturing to ensure adequate design of FDPs so that they may remain functional complication-free for longer time periods.

In this study secondary caries was observed as the least common complication in FPDs (6.5%). This is in accordance with the findings of studies conducted by Walton ^19^ and Bragger et al^20^. Bragger et al ^20^ reported the incidence of caries to be as low 2.8% and Walton ^19^ reported caries as a complication in 11% in FPDs. Other studies reported higher incidence of caries. These include studies by De Backer et al^21^ (32%), Libby et al ^22^ (38%) and Hochman et al. ^23^ The varying trend might be explained by the fact that every patient present an individualized clinical situation and maintenance / follow-up protocol. Another reason could have been the increased awareness of practitioners in selecting abutments with ideal anatomical and structural conditions for supporting the FDPs. 

Pulp pathology was seen in 7.9% of the cases. Of these 5.8% were level-1 complications and 2.2% were level-2 complications. Some international studies have also reported the incidence of pulp pathology or peri-apical problems within the same range. Schwartz et el ^24^ reported pulp pathology in 2.9% of the cases. Bragger et al ^20^ found 4.9% of the abutment teeth with peri-apical problems. Goodacre et al^10^ reported 11% of the FPDs failing because of pulp pathologies. In contrast Foster^25^, Napankangas et al ^13^ and Cheung et al^18^ reported pulp pathology as a major reason for failure of FDPS.

Periodontal disease as a biological complication was seen in 23.7% FPDs. This is in accordance with the findings of study conducted by Ikai et al^7^ on patients who did not maintain their FDPs. They reported periodontal disease as the most common reason for failure and complications in FDPs. Similarly Walton^20^ found periodontal problems in 27% FPDs. On the other hand a local study reported the incidence of periodontal disease as low as 11%. ^11^ periodontal break-down was reported in 6.8% of the FPDs by Schwartz et al^24^ and 4.6% of the FPDs by Oginni.^12^ In this study periodontal problems were mainly the result of improper maintenance of oral hygiene. A clear picture of the situation would have been apparent, had the study also considered other host-related and socio-economic factors of the study participants that certainly influence the health of the periodontal tissues.

In this study, de-cementation (41.7%) was the most common technical complication of the FPDs. A local study by Ghani and Memon^11^ also had found de-cementation as the most prominent complication in FPDs (24.8%). Oginni^12^ found cementation failure in 27.3% of the FPDs. On the contrary Garcia and Cronin^26^ found only 7% of the complications due to de-cementation. Napankangas et al^13^ reported de-cementation in 6.1% of the FPDs. 

We noted that most of the de-cemented FPDs were fitted on the abutments that had inadequate resistance and retention form. The abutments were either over-tapered or had decreased occluso-cervical dimension. Thus it would not be wrong to assume that in most cases de-cementation was the result of improper abutment preparation. The effect and contribution of the quality of the locally available luting agent could also be there and thus highlight the need for conducting a comparative bench study on these.

Aesthetic problems were seen in 20.1% FDPs. Most of them resulted from porcelain fracture. Oginni^12^ reported poor aesthetics as the most frequent cause of failure (40.5%). However a previous local study reported the incidence of aesthetic problems to be 3.3%.^11^Another study by Nappankangas^13^ reported unacceptable aesthetics in only 7% of the cases. It is to note that restoring aesthetics with MCFDPs is controlled by several factors relating to tooth preparation design, clinical judgement skills of the practitioners for proper matching of the shade tab to the adjacent teeth and the skills of the technicians in reproducing the form and shade in the laboratory.

## Conclusion

Within the limitations of the study, the following conclusion can be made:

1. The common complication in decreasing order was; de-cementation, periodontal disease, unacceptable aesthetics, pulp pathology and secondary caries.
2. Majority of the complications were in FPDs fitted in the posterior region of both the arches. However, more complications were in those fitted in the mandibular posterior region (42.4%).
3. Majority of FPDs had complications within first five years of their service life.

## Recommendations

1. Multi-centre randomized clinical controlled longitudinal trials are required to find the locally prevalent complications in FPDs.
2. The relatively greater proportion of technical complications coupled with more de-cementation events and periodontal complications highlight problems related to the clinical and technical aspects of FDP Planning and fabrication and thus emphasizing the need for acquisition and reinforcement of educational, clinical and technical skills through refresher courses and CPD programs for both clinicians and technicians.
3. Clinicians should keep themselves well-familiar of the laboratory aspects of fixed dental prostheses to properly prescribe work authorize laboratories and critically evaluate the work done for their patients,
4. The importance of oral hygiene maintenance and of regular review of patients wearing FDPs should be highlighted to patients in order to ensure long term success of FDPs.

## Notes

***Authors contribution*** **: **Both the authors contributed significantly in the conception of research idea, research protocol establishment, data collection ,analyses and manuscript preparation.
